# Type 2 diabetes subphenotypes are associated with differential outcomes after metabolic and bariatric surgery: An international multicentre retrospective cohort study

**DOI:** 10.1111/dom.70547

**Published:** 2026-03-08

**Authors:** Adisa Poljo, Jakob J. Reichl, Lars Kollmann, Piotr Kalinowski, Aleksandra Frankowska, Michał Grąt, Eleni A. Felinska, Ulrike Heger, Stefan Kopf, Donna Noeva, Christopher Tuffs, Matthias Hepprich, Eleonora Seelig, Ralph Peterli, Marko Kraljević, Jennifer M. Klasen, Beat P. Müller, Romano Schneider, Adrian T. Billeter

**Affiliations:** ^1^ Department of Visceral Surgery, Clarunis, University Digestive Health Care Center Basel St. Clara Hospital and University Hospital Basel Basel Switzerland; ^2^ Medical Faculty Johannes Kepler University Linz Linz Upper Austria Austria; ^3^ Department of Cardiology and Cardiovascular Research Institute Basel (CRIB), University Hospital Basel University of Basel Basel Switzerland; ^4^ Department of General, Visceral, Transplant, Vascular, and Pediatric Surgery University Hospital of Würzburg Würzburg Germany; ^5^ Department of General, Transplant and Liver Surgery Medical University of Warsaw Warsaw Poland; ^6^ Department of General, Visceral and Transplantation Surgery Heidelberg University Heidelberg Germany; ^7^ Department of Endocrinology, Diabetology, Metabolism and Clinical Chemistry (Internal Medicine 1) Heidelberg University Hospital Heidelberg Germany; ^8^ German Center of Diabetes Research (DZD) Neuherberg Germany; ^9^ University of Heidelberg Heidelberg Germany; ^10^ Department of General, Visceral, Thoracic, and Transplantation Surgery University of Giessen Giessen Germany; ^11^ Department of Internal Medicine and Endocrinology St Clara Hospital Basel Switzerland; ^12^ Department of Endocrinology, Diabetology and Metabolism University Hospital Basel Basel Switzerland; ^13^ Department of Clinical Research University Hospital Basel Basel Switzerland

**Keywords:** diabetes, gastric bypass, insulin resistance, metabolic surgery, sleeve gastrectomy, subphenotype

## Abstract

**Aims:**

Type 2 diabetes (T2D) is characterized by its clinical heterogeneity. Newly described T2D subphenotypes, each with distinct metabolic and co‐morbidity risk profiles, may enable a more personalized care. This study examined whether these subphenotypes can predict outcomes in patients undergoing metabolic and bariatric surgery (MBS).

**Materials and Methods:**

A total of 233 people with T2D from four clinical centres undergoing MBS were retrospectively assigned to T2D subphenotypes based on the Ahlqvist methodology. The primary outcome was T2D remission at 2 years; secondary outcomes included changes in HOMA2‐%B, HOMA2‐IR, and total body weight loss (%TWL). Intraoperative liver biopsies were evaluated for metabolic dysfunction‐associated steatotic liver disease (MASLD) and steatohepatitis (MASH).

**Results:**

All participants were classified as mild obesity‐related diabetes (MOD) (62.2%), severe insulin‐resistant diabetes (SIRD) (19.7%), or SIDD (18.1%). At 2 years, diabetes remission was lower in severe insulin‐deficient diabetes (SIDD) (36.7%) than MOD (79.3%) and SIRD (97.2%; *p* < 0.001). SIDD had lower BMI (38.3 vs. 45.2 and 43.2 kg/m^2^; *p* < 0.001) and worse beta‐cell function (HOMA2‐%B, 92.4 vs. 131.5 vs. 164; *p* = 0.010), with highest HOMA2‐IR in SIRD (6.0 ± 3.4 vs. 4.4 ± 2.8 vs. 3.6 ± 1.9; *p* < 0.001). %TWL was similar across subphenotypes. SIDD showed higher baseline MASH prevalence (60.5% vs. 46.3% vs. 46.3%).

**Conclusion:**

T2D subphenotypes respond differently to T2D remission after MBS with SIDD showing a significantly lower remission rate than MOD and SIRD at 2 years. Considering metabolic status in treatment decisions may improve patient outcomes.

## 1 INTRODUCTION

Type 2 diabetes (T2D) is an escalating global health concern and a major burden on healthcare worldwide.^1^ Metabolic and bariatric surgery (MBS) has been shown to produce substantial and long‐lasting improvements in individuals with T2D.^2^, ^3^ Bariatric surgery is recommended for adults with BMI > 30 kg/m^2^ who have inadequate glucose control on conventional treatments.^4^ Although in theory, a large proportion of people with T2D could benefit from MBS, its use in practice remains controversial due to its invasive nature.^5^ The variability in surgical outcomes highlights the complex and multifactorial characteristics of T2D.

Recent years have seen growing interest in characterizing T2D heterogeneity through data‐driven subphenotype analysis.^6^, ^7^, ^8^ Ahlqvist et al. analyzed 8980 newly diagnosed patients using glutamate decarboxylase antibodies (GADA), age at diagnosis, body mass index (BMI), HbA1c, and homoeostatic model assessment 2 estimates of beta‐cell function (HOMA2‐B) and insulin resistance (HOMA2‐IR) and identified five replicable T2D subphenotypes: severe autoimmune diabetes (SAID), severe insulin‐deficient diabetes (SIDD), severe insulin‐resistant diabetes (SIRD), mild obesity‐related diabetes (MOD), and mild age‐related diabetes (MARD).^7^ These five subphenotypes revealed significantly different patient characteristics and risk of diabetic complications.^9^ This substratification may allow earlier, more targeted treatments for those most likely to benefit, advancing precision medicine in diabetes.

Therefore, we aimed to apply the proposed subphenotype analysis to a cohort of patients with T2D undergoing MBS to evaluate its potential in predicting postoperative outcomes.

## 2 MATERIALS AND METHODS

### 2.1 Study design and patient cohort

In this retrospective, international, multi‐centre study, we assessed data‐driven T2D subphenotypes in relation to postoperative outcomes in patients undergoing MBS. This study included four high‐volume bariatric and metabolic centres from Switzerland, Germany (two centres), and Poland. The study was approved by the local Ethics Committees (2018/00356, S‐629/2013, S‐078/2010, 2019043001, KB‐0/2/2008, KB/155/2022, and KB/25/2024).

233 patients undergoing elective laparoscopic sleeve gastrectomy (SG) or Roux‐en‐Y gastric bypass (RYGB) with a diagnosis of T2D were retrospectively analyzed. In line with current treatment guidelines for obesity, the primary cohort included patients with a BMI ≥40 kg/m^2^ or a BMI of 35–40 kg/m^2^ with at least one obesity‐associated comorbidity. In addition, 16 patients from the DiaSurg1 Study^10^ were included, who had a BMI between 25 and 35 kg/m^2^, insulin‐treated T2D for at least 3 months requiring a minimum of 10 units of insulin daily, HbA1c >7.0%, and retained residual pancreatic insulin secretion as confirmed by glucagon‐stimulated C‐peptide levels >1.5 ng/mL. Patients with a confirmed history of positive diabetes‐associated autoantibodies were excluded.

Although all participants had T2D, the cohort was metabolically heterogeneous. This was addressed by classifying patients into established T2D subphenotypes that guided subsequent analyses.

### 2.2 Data collection and subphenotype allocation

Preoperatively, clinical data and blood samples were collected. Subphenotype allocation was performed via the calculator tool provided by the German Diabetes Center (DDZ) (available at: https://diabetescalculator.ddz.de/diabetescluster-en/), which was published in 2023 and described in recent studies.^11^ Variables used for clustering were sex, age at diagnosis of T2D, presence of glutamate decarboxylase antibodies (GADA), BMI, HbA1c, and homoeostatic model assessment 2 estimates of β‐cell function (HOMA2‐%B) and insulin resistance (HOMA2‐IR). According to the results after clustering patients were allocated to one of the five subphenotypes. To assess the reproducibility of the cluster structure, we performed unsupervised k‐means clustering in our cohort using five baseline variables: age at diagnosis, BMI, HbA1c, HOMA2‐%B, and HOMA2‐IR.

Additional clinical data included age at time of surgery and weight as well as standard laboratory parameters, such as insulin, C‐peptide, fasting glucose, HbA1c, platelet count, triglycerides, high‐density lipoprotein (HDL), aspartate aminotransferase (AST), and alanine aminotransaminase (ALT). Weight loss was reported as total body weight loss (TWL%). Non‐invasive tests were applied to assess the resolution of liver fibrosis. Specifically, we selected the aspartate transaminase (AST) to Platelet Ratio Index (APRI)^12^ and Fibrotic NASH Index (FNI)^13^ scores, as they are among the most suitable tools for detecting long‐term improvement of significant fibrosis following bariatric surgery.^14^

Beta‐cell function and IR were assessed using HOMA2‐%B and HOMA2‐IR, an updated model of HOMA1^15^ based on fasting glucose and C‐peptide.^16^, ^17^ Higher HOMA2‐IR (greater IR) and lower HOMA2‐%B (reduced beta‐cell function) indicate progression toward T2D.^18^ HOMA2 uses a more complex mathematical model and can be calculated with the free HOMA2 Calculator (www.OCDEM.ox.ac.uk).

The primary outcome was the difference in T2D remission rates at 2 years. T2D remission was defined following the American Diabetes Association (ADA) and European Association for the Study of Diabetes (EASD) consensus as HbA1c returning to <6.5% (<48 mmol/mol) without glucose‐lowering pharmacotherapy for at least 3 months.^19^ Improvement of T2D was defined as a clinically meaningful reduction in HbA1c (≥0.5%) and/or a decreased need for glucose‐lowering therapy, without meeting criteria for diabetes remission.^20^, ^21^ Worsening was defined as an increase in HbA1c and/or the initiation or escalation of glucose‐lowering therapy. Secondary outcomes included changes in HOMA2‐%B, HOMA2‐IR, and %TWL at 2 years.

### 2.3 Histology analysis

Liver biopsies were available for 200 patients (85.8%). Biopsies were obtained during the procedure, either as wedge biopsies from segment III or using laparoscopic biopsy forceps. Patients at increased risk of bleeding, with coagulation disorders, on anticoagulant therapy, who declined a liver biopsy, or who had incomplete laboratory data were excluded from biopsy sampling. Semi‐quantitative assessment, including fatty degeneration, inflammatory changes, and hepatocyte damage, was conducted using the NAFLD activity score (NAS) developed by Kleiner et al.^22^ by two experienced and specialized pathologists. Employing the Bedossa histological scoring system,^23^ the cohorts were then categorized into three groups based on the analysis of steatosis, ballooning, lobular inflammation, and fibrosis. Specimens were classified as “No MASLD/MASH,” “MASLD,” or “MASH” according to the Delphi consensus statement on new fatty liver disease nomenclature.^24^, ^25^

### 2.4 Statistical analysis

Continuous variables are presented as mean ± standard deviation (SD) or median with interquartile range (IQR), as appropriate. Categorical variables are summarized as counts and percentages.

Baseline characteristics were compared across subphenotypes using the Kruskal–Wallis test for continuous variables and the Chi‐square test (or Fisher's exact test when appropriate) for categorical variables. A standardized mean difference (SMD) was calculated for each comparison to assess the magnitude of imbalance between groups. Liver fibrosis was semiquantitatively evaluated using Kleiner's fibrosis score, and the description was provided without statistical comparisons.

To evaluate predictors of diabetes remission, univariate logistic regression models were fitted with 2‐year remission as the outcome. Baseline predictors assessed individually included age at diagnosis, body mass index (BMI), HbA1c, HOMA2‐%B, HOMA2‐IR, and diabetes duration. Results are reported as odds ratios with 95% confidence intervals.

A multivariable logistic regression including subphenotype assignment and all baseline variables was used to determine whether subphenotype membership provided prognostic information beyond individual metabolic measures. Sensitivity analyses using de novo subphenotype assignments were performed to assess robustness. All variables were standardized to zero mean and unit variance.

The optimal number of clusters was determined using the silhouette method, gap statistic, and elbow method, and cluster stability was evaluated with 100 bootstrap resamples using the Jaccard index. Subphenotype assignments were then used to repeat outcome analyses as a sensitivity check. Spearman rank correlations were used to account for non‐normal distributions and were performed separately within each subphenotype to examine subphenotype‐specific associations between weight loss and metabolic changes.

Statistical significance was set at a two‐sided *p*‐value <0.05. All analyses were performed using R (version 4.4.0; R Foundation for Statistical Computing, Vienna, Austria), using the gtsummary, tableone and ggplot2 packages.

## 3 RESULTS

### 3.1 Baseline characteristics

A total of 233 patients with T2D were included in the study and classified into three subphenotypes: MOD (*n* = 145, 62.2%), SIRD (*n* = 46, 19.8%), and SIDD (*n* = 42, 18.0%). No patients were allocated to the SAID or MARD subphenotype. Significant heterogeneity in demographic and metabolic characteristics was observed among the subphenotypes. Baseline characteristics and 2‐year outcomes are presented in Table 1, while interim 1‐year data are provided in Table S1.

**TABLE 1:** Baseline characteristics and 2‐year outcomes based on T2D subphenotypes.

Variable	Overall	MOD	SIRD	SIDD	*p*‐value^b^
	*N* = 233^a^	*N* = 145^a^	*N* = 46^a^	*N* = 42^a^	
Age at surgery (years)	49.2 ± 9.6	47.4 ± 9.6	51.0 ± 8.7	53.5 ± 8.9	**<0.001**
Age at T2D diagnosis (years)	43.4 ± 9.7	42.1 ± 9.6	48.0 ± 8.3	42.9 ± 10.1	**<0.001**
T2D duration (years)	5.8 ± 6.2	5.3 ± 5.6	3.0 ± 3.4	10.6 ± 7.9	**<0.001**
Sex (%)					0.70
Male	107 (45.9%)	67 (46.2%)	19 (41.3%)	21 (50.0%)	
Female	126 (54.1%)	78 (53.8%)	27 (58.7%)	21 (50.0%)	
Procedure type (%)					**0.005**
SG	148 (63.5%)	103 (71.0%)	26 (56.5%)	19 (45.2%)	
RYGB	85 (36.5%)	42 (29.0%)	20 (43.5%)	23 (54.8%)	
Insulin‐treated T2D (%)	59 (25.3%)	28 (19.3%)	6 (13.0%)	25 (59.5%)	**<0.001**
BMI (kg/m^2^)					
Baseline	43.6 ± 7.0	45.2 ± 7.0	43.2 ± 6.0	38.3 ± 5.0	**<0.001**
2 years	31.8 ± 6.0	32.6 ± 5.7	31.9 ± 6.2	28.4 ± 5.6	**0.001**
HbA1c (%)					
Baseline	7.6 ± 1.8	7.3 ± 1.6	6.6 ± 1.1	9.6 ± 1.7	**<0.001**
2 years	6.0 ± 1.0	5.9 ± 0.9	5.5 ± 0.4	6.9 ± 1.1	**<0.001**
HOMA2‐%B					
Baseline	116.3 ± 75.1	109.0 ± 58.1	200.2 ± 76.6	49.7 ± 31.4	**<0.001**
2 years	133.0 ± 46.8	131.5 ± 36.2	164.7 ± 52.6	92.4 ± 54.7	**0.01**
HOMA2‐IR					
Baseline	4.2 ± 2.6	3.6 ± 1.9	6.0 ± 3.4	4.4 ± 2.8	**<0.001**
2 years	2.0 ± 0.7	1.8 ± 0.6	2.4 ± 0.6	2.0 ± 1.1	**0.01**
T2D status at 2 years					**<0.001**
Worsened	10 (5.3%)	8 (6.6%)	0 (0.0%)	2 (6.7%)	
Unchanged	6 (3.2%)	4 (3.3%)	0 (0.0%)	2 (6.7%)	
Improved	29 (15.5%)	13 (10.7%)	1 (2.8%)	15 (50.0%)	
Remission	142 (75.9%)	96 (79.3%)	35 (97.2%)	11 (36.7%)	
%TWL					
2 years	26.0 ± 10.0	26.4 ± 10.0	26.0 ± 8.8	24.5 ± 11.1	0.80
Blood platelets (G/L)					
Baseline	251.5 ± 75.7	255.4 ± 67.0	258.5 ± 97.8	230.5 ± 75.8	0.20
2 years	238.2 ± 68.0	245.6 ± 69.0	235.9 ± 60.6	212.7 ± 68.4	0.07
AST (U/L)					
Baseline	33.8 ± 22.2	34.4 ± 22.2	32.4 ± 25.5	33.3 ± 18.6	0.80
2 years	23.1 ± 6.9	23.9 ± 7.5	22.0 ± 5.3	21.4 ± 5.9	0.30
ALT (U/L)					
Baseline	42.8 ± 27.0	43.9 ± 28.5	38.1 ± 27.0	43.8 ± 20.7	0.08
2 years	24.0 ± 9.4	24.5 ± 9.1	22.6 ± 10.0	24.3 ± 9.6	0.40
APRI‐score					
Baseline	0.4 ± 0.3	0.4 ± 0.3	0.4 ± 0.4	0.4 ± 0.3	0.20
2 years	0.3 ± 0.1	0.3 ± 0.1	0.2 ± 0.1	0.3 ± 0.2	0.80
HDL (mg/dL)					
Baseline	42.3 ± 11.8	43.8 ± 12.1	39.7 ± 10.4	39.8 ± 11.3	0.11
2 years	59.4 ± 14.3	61.6 ± 14.3	57.4 ± 13.7	53.3 ± 13.5	**0.004**
GFR (mL/min/1.73)					
Baseline	92.9 ± 20.1	93.2 ± 19.7	89.8 ± 18.4	95.3 ± 23.1	0.60
2 years	93.1 ± 19.2	92.0 ± 20.3	91.1 ± 15.1	100.3 ± 18.7	0.11
Creatinine (mg/dL)					
Baseline	0.8 ± 0.2	0.8 ± 0.2	0.9 ± 0.3	0.8 ± 0.3	0.60
2 years	0.8 ± 0.2	0.8 ± 0.2	0.8 ± 0.2	0.7 ± 0.2	0.11
NAS‐score at baseline	4.74 ± 1.88	4.73 ± 1.94	4.49 ± 1.85	5.03 ± 1.75	0.50
FNI					
Baseline	0.4 ± 0.3	0.4 ± 0.3	0.3 ± 0.2	0.6 ± 0.3	**<0.001**
2 years	0.1 ± 0.1	0.1 ± 0.1	0.1 ± 0.1	0.2 ± 0.2	**0.002**
Liver histology grading					0.60
No MASLD/MASH	28 (14.0%)	18 (14.9%)	6 (14.6%)	4 (10.5%)	
MASLD	74 (37.0%)	47 (38.8%)	16 (39.0%)	11 (28.9%)	
MASH	98 (49.0%)	56 (46.3%)	19 (46.3%)	23 (60.5%)	
Fibrosis score					0.50
0	50 (25.5%)	33 (28.0%)	11 (26.8%)	6 (16.2%)	
1	66 (33.7%)	42 (35.6%)	14 (34.1%)	10 (27.0%)	
2	62 (31.6%)	33 (28.0%)	11 (26.8%)	18 (48.6%)	
3	10 (5.1%)	6 (5.1%)	3 (7.3%)	1 (2.7%)	
4	8 (4.1%)	4 (3.4%)	2 (4.9%)	2 (5.4%)	

*Note*: Bold values indicate statistically significant differences between subphenotypes (*p* < 0.05).

Abbreviations: ALT, alanine aminotransferase; APRI, aspartate aminotransferase to platelet ratio index; AST, aspartate aminotransferase; BMI, body mass index; FNI, Fibrotic NASH index; GFR, glomerular filtration rate; HDL, high‐density lipoprotein; HOMA2‐%B, homeostatic model assessment 2 of beta‐cell function; HOMA2‐IR, homeostatic model assessment 2 of insulin resistance; MASLD, metabolic dysfunction‐associated steatotic liver disease; MASH, metabolic dysfunction‐associated steatohepatitis; MOD, mild obesity‐related diabetes; NAS, NAFLD activity score; RYGB, Roux‐en‐Y gastric bypass; SG, sleeve gastrectomy; SIDD, severe insulin‐deficient diabetes; SIRD, severe insulin‐resistant diabetes; T2D, type 2 diabetes; %TWL, percentage of total weight loss.

^a^ Mean ± SD; *n* (%).

^b^ Kruskal–Wallis rank sum test; Pearson's Chi‐squared test.

At surgery, SIDD patients were oldest (53.5 ± 8.9 years), followed by SIRD (51.0 ± 8.7) and MOD (47.4 ± 9.6 years; *p* < 0.001). Baseline BMI was highest in MOD (45.2 ± 7.0 kg/m^2^), lower in SIRD (43.2 ± 6.0 kg/m^2^), and lowest in SIDD (38.3 ± 5.0 kg/m^2^; *p* < 0.001).

### 3.2 Diabetes status and remission rate after surgery

Age at diabetes diagnosis was later in SIRD (48.0 ± 8.3 years) than in MOD (42.1 ± 9.6 years) and SIDD (42.9 ± 10.1 years; *p* < 0.001). T2D duration differed by subphenotype (*p* < 0.001), longest in SIDD (10.6 ± 7.9 years), intermediate in MOD (5.3 ± 5.6 years), and shortest in SIRD (3.0 ± 3.4 years).

Follow‐up for T2D remission at 2 years was available for 80.3% of patients (*n* = 187) overall. By diabetes sub‐phenotype, remission rates were available for 84.1% in MOD (122/145), 78.3% in SIRD (36/46), and 71.4% in SIDD (30/42).

At 2 year postoperatively, remission of T2D was achieved in 79.3% of MOD, 97.2% of SIRD, and 36.7% of SIDD patients (*p* < 0.001). An additional 10.7% of MOD, 2.8% of SIRD, and 50.0% of SIDD showed improvement without full remission. Notably, no SIRD patients experienced worsening of diabetes compared with 6.6% of MOD and 6.7% of SIDD.

In univariate logistic regression, baseline HbA1c (OR 0.60, 95% CI 0.48–0.72, *p* < 0.001), HOMA2‐%B (OR 1.02, 95% CI 1.01–1.03, *p* < 0.001), and diabetes duration (OR 0.92 per year, 95% CI 0.87–0.97, *p* = 0.002) were significantly associated with 2‐year diabetes remission. Baseline BMI showed a nonsignificant trend toward higher remission rates (OR 1.06, *p* = 0.096), whereas age at diagnosis and HOMA2‐IR (OR 1.08, 95% CI 0.92–1.30, *p* = 0.38) were not significantly associated with remission.

In multivariable logistic regression including subphenotype, age at diagnosis, BMI, HbA1c, HOMA2‐%B, HOMA2‐IR, and diabetes duration, only age at diagnosis remained independently associated with remission (OR 0.95 per year, 95% CI 0.90–0.99, *p* = 0.035); subphenotype and other metabolic variables were no longer significant. This attenuation suggests that the prognostic information conveyed by subphenotype assignment is largely captured by established clinical and metabolic factors, rather than indicating an independent effect of subphenotype membership itself.

### 3.3 Diabetes medication use at baseline and follow‐up

Baseline glucose‐lowering therapy differed across diabetes clusters (Tables S2 and S3). Metformin was the most commonly prescribed agent in all clusters, with greater use in MOD and SIRD and lower use in SIDD. Insulin use was highest in SIDD, both in terms of the proportion of patients receiving insulin and the overall number of glucose‐lowering drug classes used. Use of GLP‐1 receptor agonists, SGLT2 inhibitors, and DPP‐4 inhibitors was relatively low across all clusters.

At 2 years, medication use declined markedly in all clusters, although SIDD retained the highest rates of oral antidiabetic and insulin therapy. Notably, many patients were not receiving glucose‐lowering treatment, including some who did not meet formal remission criteria (Table S4).

### 3.4 Glycaemic control and insulin dependence

Baseline HbA1c was lowest in SIRD (6.6 ± 1.1%), intermediate in MOD (7.3 ± 1.6%), and highest in SIDD (9.6 ± 1.7%; *p* < 0.001). Correspondingly, insulin treatment was most prevalent in SIDD (59.5%), compared with 19.3% in MOD and 13.0% in SIRD (*p* < 0.001). Following MBS, HbA1c decreased significantly in all groups. At 2 years, HbA1c values were 5.9 ± 0.9% (MOD), 5.5 ± 0.4% (SIRD), and 6.9 ± 1.1% (SIDD; *p* < 0.001).

### 3.5 Beta‐cell function and insulin resistance

Despite similar weight loss, metabolic improvement varied across subphenotypes. At baseline, SIRD had the highest beta‐cell function (HOMA2‐%B 200.2 ± 76.6), followed by MOD (109.0 ± 58.1), and SIDD (49.7 ± 31.4; *p* < 0.001), and also the greatest IR (HOMA2‐IR 6.0 ± 3.4 vs. 4.4 ± 2.8 in SIDD and 3.6 ± 1.9 in MOD; *p* < 0.001). At 2 years, HOMA2‐%B remained highest in SIRD (164.7 ± 52.6), intermediate in MOD (131.5 ± 36.2), and lowest in SIDD (92.4 ± 54.7; *p* = 0.010). HOMA2‐IR improved in all subphenotypes at 2 years (1.8 ± 0.6 MOD, 2.4 ± 0.6 SIRD, 2.0 ± 1.1 SIDD; *p* = 0.009). Changes in BMI, HOMA2‐IR, HOMA2‐%B, and HbA1c are shown in Figure S1A–D.

### 3.6 HOMA2‐indices according to subphenotypes and T2D remission status

In the MOD subphenotype, patients who achieved T2D remission exhibited significantly higher HOMA2‐%B values at baseline (108.3 ± 43.5 vs. 79.0 ± 46.8, *p* = 0.002), at 1 year (133.6 ± 61.3 vs. 84.0 ± 36.7, *p* = 0.02), and at 2 years (135.4 ± 34.0 vs. 86.1 ± 24.9, *p* = 0.001). In the other subphenotypes, no significant differences were observed in HOMA2‐%B or HOMA2‐IR between patients with and without T2D remission. Additional details are provided in Table S5 and Figure S2A,B.

### 3.7 HOMA2‐indices according to subphenotype and surgery type

In the MOD subphenotype, HOMA2‐%B or HOMA2‐IR were comparable between procedure types, although a trend toward lower IR was noted after RYGB at 2 years (*p* = 0.102). In the SIRD subphenotype, HOMA2‐IR was significantly lower at 2 years following SG (*p* = 0.04). In the SIDD subphenotype, no significant differences were detected between procedure types for either variable at any time point; however, HOMA2‐%B appeared higher after SG at 2 years (101.9 ± 57.7 vs. 54.6 ± 9.8), though this difference was not statistically significant, likely due to sample size limitations. Further details are available in Table S6 and Figure S2C,D.

### 3.8 DiaSurg1 versus non‐DiaSurg1 patients in the SIDD subphenotype

We performed a subgroup analysis of DiaSurg1 and non‐DiaSurg1 patients within the SIDD subphenotype (Table S7). Of 16 DiaSurg1 patients, 14 were SIDD and 2 SIRD; thus, 42 SIDD patients were included (14 DiaSurg1, 28 non‐DiaSurg1).

DiaSurg1 patients differed from non‐DiaSurg1 in baseline and postoperative characteristics. In accordance with the study design, all DiaSurg1 patients underwent RYGB compared with 32% of non‐DiaSurg1 SIDD patients. DiaSurg1 patients were older (58 vs. 51 years) and had lower BMI at surgery (32.8 vs. 41 kg/m^2^). Although non‐DiaSurg1 patients had a higher BMI, they were still appropriately classified as the SIDD subphenotype, as evidenced by poorer glycaemic control and more severe metabolic dysfunction at baseline, including higher HbA1c (10.16% vs. 8.37%), lower beta‐cell function (HOMA2‐%B 39 vs. 71), and slightly greater IR (HOMA2‐IR 4.0 vs. 3.78).

At 2 years, DiaSurg1 patients had higher HbA1c (7.49% vs. 6.68%) and lower T2D remission (11% vs. 48%).

### 3.9 Weight loss

%TWL data were available for 95.2% (*n* = 222) at 1 year and 86.3% (*n* = 201) at 2 years. All subphenotypes achieved substantial and similar weight loss. At 2 years, %TWL was 26.4 ± 10.0% (MOD), 26.0 ± 8.8% (SIRD), and 24.5 ± 11.1% (SIDD; *p* = 0.8). Across subphenotypes, %TWL tended to be higher in patients achieving T2D remission: MOD 27.0% vs. 23.2% (*p* = 0.08), SIDD 29.8% vs. 21.3% (*p* = 0.004), and SIRD 27.1% vs. 19.2% (*p* = 0.05). There were no significant differences in %TWL between surgery types (Table S8).

Within‐subphenotype analyses showed weak correlations between 2‐year weight loss and glycaemic/metabolic changes (Figure S3). MOD showed no significant associations except a borderline inverse correlation with HOMA2‐IR. SIRD had moderate but non‐significant inverse correlations with HOMA2‐IR and HOMA2‐%B, likely due to limited sample size. SIDD showed no meaningful correlations, suggesting glycaemic improvements were largely independent of weight loss.

### 3.10 Liver and renal function

Intraoperative liver histology was available for 200 patients (85.8%), including 83.4% of MOD (121/145), 89.1% of SIRD (41/46), and 90.5% of SIDD (38/42). Histologic findings were similar across sub‐phenotypes, with MASLD in 38.8% (MOD), 39.0% (SIRD), and 10.5% (SIDD), and MASH in 46.3%, 46.3%, and 60.5%, respectively. Baseline FNI was highest in SIDD (0.6 ± 0.3; predicted 60% probability of fibrotic MASH) versus MOD (0.4 ± 0.3) and SIRD (0.3 ± 0.2; *p* < 0.001). FNI decreased postoperatively in all subphenotypes but remained elevated in SIDD at 2 years (0.2 ± 0.2; *p* = 0.002). Liver enzymes (AST, ALT) and APRI scores did not differ between subphenotypes over time.

Ordinal logistic regression of liver histology (no MASLD, MASLD, MASH), adjusted for age at diagnosis, diabetes duration, and BMI, showed HOMA2‐IR was independently associated with greater liver disease severity (OR 1.21, 95% CI 1.07–1.40, *p* = 0.006), while HOMA2‐%B was inversely associated (OR 0.994, 95% CI 0.990–0.997, *p* = 0.001). Age, BMI, and diabetes duration were not independently significant, though diabetes duration improved overall model fit.

Renal function was generally preserved. Mean eGFR was similar at baseline (*p* = 0.6) but transiently higher in SIDD at 2 years (100.3 ± 18.7 vs. 92.0 ± 20.3 vs. 91.1 ± 15.1; *p* = 0.11), possibly reflecting hyperfiltration due to suboptimal glycaemic control.

### 3.11 De novo clustering for reproducibility of subphenotypes

To evaluate the robustness of our findings, we performed unsupervised k‐means clustering using age at diagnosis, BMI, HbA1c, HOMA2‐%B, and HOMA2‐IR. Three metabolically distinct subphenotypes (*n* = 115, 58, 60) were identified, consistent with the patterns observed using the external DDZ clustering tool (Figures S4 and S5). Concordance analysis demonstrated substantial overlap between de novo and predefined subphenotypes (Table S9). De novo subphenotype 1 showed high concordance with MOD, while subphenotype 3 aligned predominantly with SIRD. De novo subphenotype 2 exhibited partial overlap with MOD and SIDD, consistent with a more insulin‐deficient phenotype and reflecting expected heterogeneity due to differences in clustering variables.

T2D remission at 2 years differed substantially across groups, with subphenotype 2 exhibiting the lowest remission rate (53.8%), whereas subphenotype 1 and 3 showed high remission rates (84.3% and 91.7%, respectively; *p* < 0.01). Notably, subphenotype 2 displayed features consistent with an insulin‐deficient phenotype, mirroring the findings observed using the predefined subphenotype classification (Tables S10 and S11).

## 4 DISCUSSION

In this international, multicentre observational study, we evaluated T2D subphenotypes, as proposed by Ahlqvist et al.,^7^ in patients undergoing MBS. Our study contributes to the growing body of evidence on MBS, especially in the context of a rising number of patients referred for metabolic and bariatric care. We report the following main findings:

First, we found that the proposed subphenotypes were reproducible in this patient population, with distinct differences between all three subphenotypes. Second, patients in the SIDD group expressed worse baseline diabetes control compared to the other groups, showing the lowest beta‐cell function while also having the lowest BMI at the time of surgery. Third, SIDD patients had the lowest T2D remission rates up to the 2‐year follow‐up despite comparable weight loss after MBS. Importantly, however, this group still demonstrated marked metabolic improvements following surgery, underscoring the substantial benefit of MBS in this high‐risk population, particularly given their lower BMI.

**FIGURE 1:**
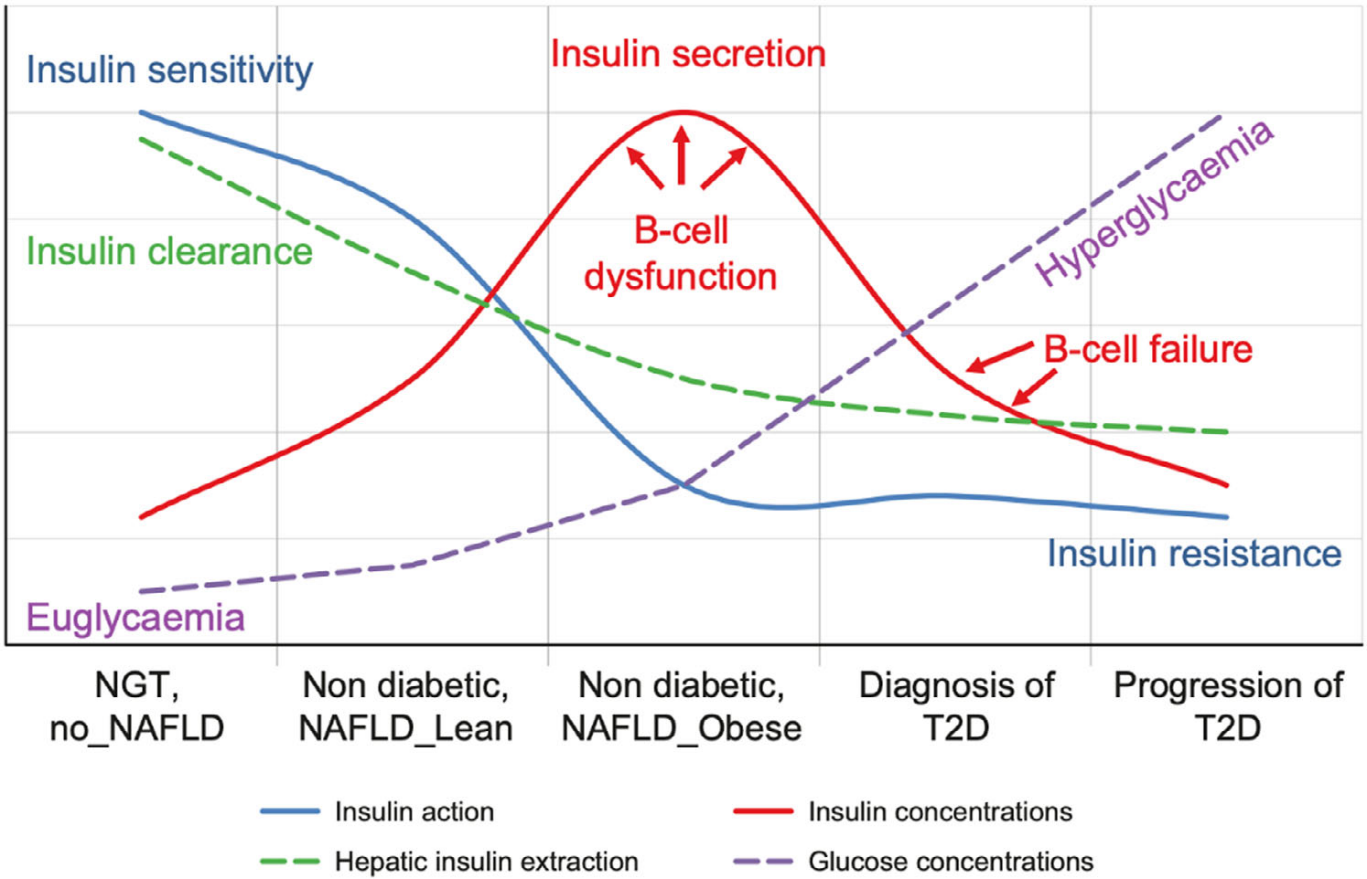
Natural history of T2D in the context of NAFLD (used with permission from Ref. 26). IR, insulin resistance; NAFLD, non‐alcoholic fatty liver disease; NGT, normal glucose tolerance; T2D, type 2 diabetes.

As demonstrated in Figure 1,^26^ T2D‐related hyperglycaemia initially triggers beta‐cell overcompensation and high insulin production, which eventually declines over time. Patients with SIRD exhibited high insulin secretion and IR pre‐surgery, reflected in elevated HOMA2‐%B and HOMA2‐IR. Post‐surgery, both insulin production and IR decreased, indicating improved insulin sensitivity and reduced need for beta‐cell overcompensation. The drop in HOMA2‐%B should be interpreted alongside IR improvement, reflecting amelioration of hyperinsulinemia. In contrast, SIDD patients had markedly lower beta‐cell function at baseline; although HOMA2‐%B slightly increased after surgery, it remained lower than other subphenotypes, indicating at least a partial recovery of beta‐cell function through MBS.

Based on these findings, we hypothesize that patients in the SIDD group may exhibit more advanced beta‐cell dysfunction, which could contribute to their poorer glycaemic control at baseline and lower remission rates after MBS, consistent with their longer disease duration. The association between T2D duration and glycaemic control has been described in prior literature,^27^, ^28^, ^29^ and longer disease duration has been associated with increased cardiovascular risk and mortality.^30^, ^31^

Raverdy et al.^32^ specifically investigated the responsiveness of the SIRD subtype to MBS, hypothesizing that SIRD patients may derive greater metabolic benefit compared with other subtypes. Their findings align with ours, as SIRD patients in both studies achieved the highest T2D remission rates at 1 year (81.3%, 90.0% in^32^ and 90.7% in our study) postoperatively. Additionally T2D remission rates in SIDD patients were low and comparable in both studies (12.5%, 36.0% in^32^ and 38.1%).

Importantly, an unsupervised de novo clustering approach, independent of the original classification scheme, reproduced the central finding that an insulin‐deficient phenotype is associated with reduced likelihood of T2D remission following MBS.

The study's stringent T2D remission criterion required HbA1c <6.5% without medication for ≥3 months. SIDD patients started with higher baseline HbA1c (9.6%), and although only 36.7% achieved remission at 2 years, 50.0% of patients experienced clinically meaningful improvement in glycaemic control. The average reduction in HbA1c to 6.9%–7.0% represents a substantial improvement associated with lower risk of microvascular and macrovascular complications and should be considered a clinically significant benefit of MBS, even in the absence of formal remission.^33^, ^34^

Another important finding was the different proportions of SG and RYGB across subphenotypes. SIDD patients more often underwent RYGB over SG, which was the opposite in the MOD group. RYGB has demonstrated superior long‐term outcomes compared to SG.^35^, ^36^, ^37^ Therefore, these differences of the defining surgery may have an influence on metabolic outcomes, especially with longer follow‐up as SG and RYGB differences in weight loss become significant after 5 years.^36^, ^37^, ^38^, ^39^ However, the choice of procedure likely also reflects clinical practice at the study sites, where RYGB is commonly preferred for patients with higher metabolic burden.^40^, ^41^, ^42^, ^43^

Consistent with similar %TWL across subphenotypes, these results suggest that MBS benefits are not solely weight loss‐dependent. While overall weight loss was comparable, T2D remission varied, highlighting both weight‐dependent and weight‐independent mechanisms. Early mechanistic studies show that procedures such as RYGB can normalize fasting glucose and improve insulin sensitivity within days to weeks after surgery, preceding substantial weight loss and are accompanied by enhanced incretin responses, including increased GLP‐1‐mediated beta‐cell responsiveness.^44^ Furthermore, recent evidence from non‐surgical interventions indicates that remission of dysglycaemia can occur even without weight loss, likely mediated by improvements in insulin sensitivity, adipose tissue distribution, and other metabolic adaptations.^45^

These findings suggest surgery‐induced metabolic changes beyond weight loss contribute to T2D remission and explain why %TWL alone does not consistently predict remission across subphenotypes.

As described in prior research,^46^ both high IR and insufficient beta‐cell compensation contribute to MASH development. Consistently, SIDD patients had the highest MASH rates. The decline in FNI over 2 years was similar across groups, supporting prior reports of MASH remission after metabolic surgery.^47^, ^48^ SIDD patients had a baseline FNI of 0.6, corresponding to a 60% predicted probability of fibrotic NASH, which matched the 60.5% histological prevalence, highlighting FNI's value as a non‐invasive MASH marker. Our observation that MASH prevalence was highest in the SIDD subgroup, characterized by severe beta‐cell dysfunction, suggests that beta‐cell failure may accelerate progression from steatosis to steatohepatitis, complementing the established role of IR in MASH pathogenesis.^49^ This is supported by our ordinal logistic regression analysis, in which both higher IR and lower beta‐cell function were independently associated with increasing liver disease severity.

Importantly, our findings differ from previous reports on MASLD and MASH. Ahlqvist et al.^7^ reported the highest MASLD rates in SIRD (24.1%) without distinguishing MASH, whereas in our cohort MASLD was most common in SIRD (39.0%) and MASH highest in SIDD (60.5%). The reasons behind these differences may be twofold. First, we employed histologic diagnosis using liver biopsy at baseline in every patient, whereas MASLD in^7^ was defined by the presence of two pathological ALT measurements and a BMI greater than 28 kg/m^2^ and no validated score was used. Second, our population consists of patients eligible for MBS, while the original paper was based on an unfiltered population of patients with de novo T2D.

Similarly, Zaharia et al.^9^ assessed liver involvement using hepatocellular lipid content by magnetic resonance spectroscopy and non‐invasive fibrosis scores, finding MASLD‐related traits most pronounced in SIRD, consistent with our MASLD results (39.0% in SIRD, 38.8% in MOD, 28.9% in SIDD). However, MASH represents a histological diagnosis defined by hepatocellular ballooning and inflammation, which cannot be captured by imaging or non‐invasive scores. Thus, differences between subphenotypes with respect to steatosis burden (MASLD) versus histologically defined steatohepatitis (MASH) are expected and highlight the added value of biopsy‐based assessment.

We hypothesize that the apparent lack of documented precursor states in SIDD may reflect accelerated disease rather than biological implausibility. Rapid hyperglycaemia, metabolic decline, and beta‐cell failure may drive fast progression from steatosis to steatohepatitis and fibrosis, shortening the detectable phase of isolated steatosis. Conventional markers (BMI, triglycerides, IR) may underestimate liver risk, as hyperglycaemia and lipolysis independently promote hepatic inflammation and fibrosis. Limited MASLD screening and delayed diagnosis, especially in non‐severely obese individuals, may further obscure early disease.

Lower T2D remission in SIDD, despite improved insulin resistance and partial beta‐cell recovery, suggests it is a distinct metabolic phenotype rather than advanced T2D. This idea is supported by another recent work from Raverdy et al.,^50^ who identified MASLD subtypes with similar liver histology but different metabolic profiles. Their ‘cardiometabolic’ MASLD subphenotype, characterized by dysglycaemia, high triglycerides, and frequent insulin use, mirrors SIDD features. This implies limited remission may reflect inherent vulnerabilities, such as impaired beta‐cell resilience or altered glucose–lipid pathways, rather than weight loss or disease duration alone. Raverdy et al. also reported that GLP‐1 receptor agonists may be particularly effective in this subtype, indicating SIDD patients could benefit from a tailored approach combining MBS with incretin‐based therapies.

Finally, the importance of discovering patients with high metabolic burden cannot be overstated. This is especially important in patients suffering from obesity, where current clinical practice demonstrates an overreliance on BMI alone. Our findings underline that this reductionistic and one‐dimensional approach of assessing a patient solely on BMI inadequately reflects the multifactorial nature of obesity.^51^ Especially in the context of indication for MBS in patients with a BMI of 30–35 kg/m^2^ and poorly controlled T2D, early MBS may lead to better outcomes in those with SIDD. Using accessible tools, such as the subphenotypes proposed by Ahlqvist et al., can help identify high‐risk patients, counter therapeutic nihilism, and enable individualized, patient‐centred care. Such stratification is also valuable for designing future RCTs and registries.^52^, ^53^

## 5 LIMITATIONS

This study has several limitations. Its retrospective design may limit generalizability and introduce selection bias, highlighting the need for prospective validation. Procedure selection varied across centres and was not standardized, and data on key cardiovascular and metabolic comorbidities were unavailable, which could have influenced outcomes. Differences in the proportions of surgical procedures may also have impacted results. Interpretation of HOMA‐derived indices should be cautious in patients receiving insulin, as exogenous insulin suppresses endogenous C‐peptide secretion. Additionally, the relatively long diabetes duration in our cohort may affect subphenotype stability. SIDD prevalence typically declines after approximately 5 years, meaning current glycaemic control may reflect treatment effects rather than underlying pathophysiology, potentially attenuating or shifting subphenotype characteristics. Follow‐up completeness varied across subphenotypes, with T2D remission status at 2 years available in 71%–84% of patients and liver histology in 83%–91%, which may introduce selection bias and limit generalizability. Finally, the study was not powered to detect differences in T2D remission between surgical procedures. To detect a 10‐percentage point difference in T2D remission rates between surgical procedures, approximately 700 patients with T2D would need to be enrolled.

Despite these limitations, our study provides invaluable real‐world data from bariatric high‐volume centres, including the highest number of post‐surgical SIDD patients reported to date and offering important insights into subphenotype‐specific outcomes after metabolic bariatric surgery.

## 6 CONCLUSION

Patients with SIDD were less likely to achieve full T2D remission 2 years after MBS. Nevertheless, half of SIDD patients showed meaningful improvements in glycaemic control, including substantial HbA1c reduction and partial beta‐cell recovery, demonstrating that surgery provides significant clinical benefit even in this high‐risk subgroup. These findings highlight the value of assessing metabolic profile alongside BMI and support a personalized approach to optimize outcomes for all T2D subphenotypes.

## FUNDING INFORMATION

There was no funding for this study.

## CONFLICT OF INTEREST STATEMENT

The authors have no conflicts of interest to declare.

## ETHICS STATEMENT

The authors are accountable for all aspects of the work in ensuring that questions related to the accuracy or integrity of any part of the work are appropriately investigated and resolved.

The trial was conducted in accordance with the Declaration of Helsinki (as revised in 2013). The study was approved by the local Ethics Committees (2018/00356, S‐629/2013, S‐078/2010, 2019043001, KB‐0/2/2008, KB/155/2022, and KB/25/2024) and informed consent was taken from all individual participants.

## Supporting information


**Appendix S1:** Supporting information.

## Data Availability

Data is available upon reasonable request.
