# Supplementary material for: Type 2 diabetes subphenotypes are associated with differential outcomes after metabolic and bariatric surgery: An international multicentre retrospective cohort study
Source: Diabetes Obes Metab. 2026 Mar 8;28(5):3681–91. doi: 10.1111/dom.70547 (PMC13071240; doi:10.1111/dom.70547)
Supplement: Supplementary file 1 — Appendix S1: Supporting information. [file DOM-28-3681-s001.pdf]

SUPPLEMENTARY MATERIAL

Type 2 diabetes subphenotypes are associated with differential outcomes after metabolic and bariatric surgery: an international multicenter retrospective cohort study

Adisa Poljo, MD, Jakob J. Reichl, MD, Lars Kollmann, MD, Piotr Kalinowski, MD, Aleksandra Frankowska, MD, Michał Grąt, MD, Eleni A. Felinska, MD, Ulrike Heger, MD, Stefan Kopf, MD,Donna Noeva, MD, Christopher Tuffs, MD, Matthias Hepprich, MD, Eleonora Seelig, MD, Ralph Peterli, MD, Marko Kraljević, MD, Jennifer M. Klasen, MD, PhD, Beat P. Müller, MD, Romano Schneider, MD, Adrian T. Billeter, MD, PhD

Table Of Contents

Supplementary Figure S1 A-D: Changes of BMI (A), HbA1c (B), HOMA2-%B (C) and HOMA2-IR (D) between baseline and two years follow-up. 2

Supplementary Figure S2. HOMA2- indices over time by Subphenotype and T2D remission status (A, B) and type of surgery (C, D).... 3

Supplementary Figure S3. Correlation between percent total weight loss at 2 years and changes in glycaemic and metabolic variables stratified by diabetes subphenotypes. 4

Supplementary Figure S4: Determination of the optimal number of clusters using the Silhouette width (A), gap statistic (B), and within-cluster sum of squares (elbow method)(C). 5

Supplementary Figure S5: Principal component analysis (PCA) of de novo clusters. PCA visualization demonstrates separation of the three de novo clusters based on scaled metabolic variables, with ellipses indicating cluster dispersion. 6

Supplementary Table S1: One-year outcomes based on T2D-subphenotypes.... 7

Supplementary Table S2: Baseline diabetes medication by subphenotype 9

Supplementary Table S3: Number of diabetes medications and insulin use at baseline 10

Supplementary Table S4: Diabetes medications and insulin use at 2-year follow-up 11

Supplementary Table S5: HOMA2-indices by subphenotype and T2D remission status....12

Supplementary Table S6: HOMA2-indices by subphenotype and surgery type 13

Supplementary Table S7. Baseline Characteristics and Two-Year Outcomes Stratified by DiaSurg1 Status Within the SIDD Cluster....14

Supplementary Table S8: %TWL by subphenotype and surgery type....15

Supplementary Table S9: Crosstable demonstrating concordance between original subphenotypes and de-novo subphenotype....16

Supplementary Table S10: Metabolic characteristics of de-novo subphenotypes 17

Supplementary Table S11: Diabetes remission rates at two years between de-novo subphenotypes. 18

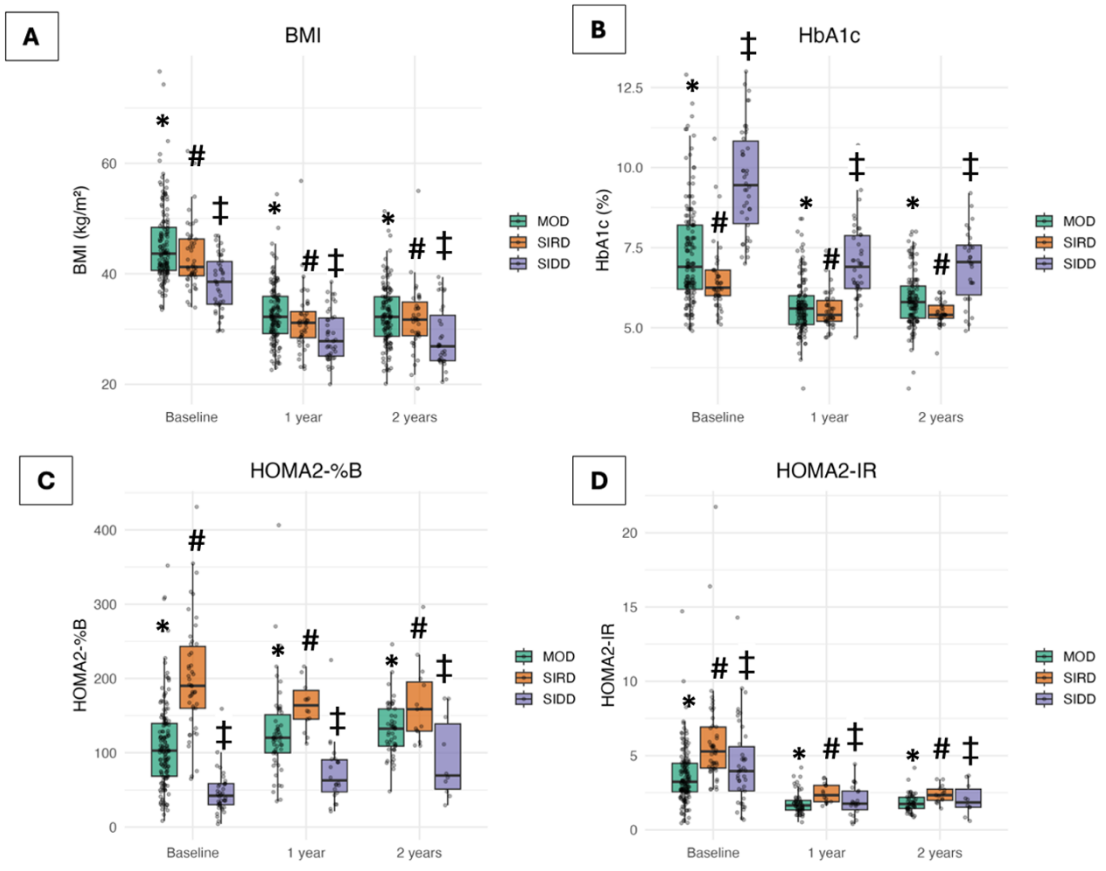

Supplementary Figure S1 A-D: Changes of BMI (A), HbA1c (B), HOMA2-%B (C) and HOMA2-IR (D) between baseline and two years follow-up.

Abbreviations: BMI, body mass index; HOMA2-%B, homeostatic model assessment 2 of beta-cell function; HOMA2-IR, homeostatic model assessment 2 of insulin resistance; MOD, mild obesity-related diabetes; SIDD, severe insulin-deficient diabetes; SIRD, severe insulin-resistant diabetes
*, $p<0.05$ at baseline between subphenotypes
#, $p<0.05$ at 1 year between subphenotypes
‡, $p<0.05$ at 2 years between subphenotypes

SUPPLEMENTARY MATERIAL

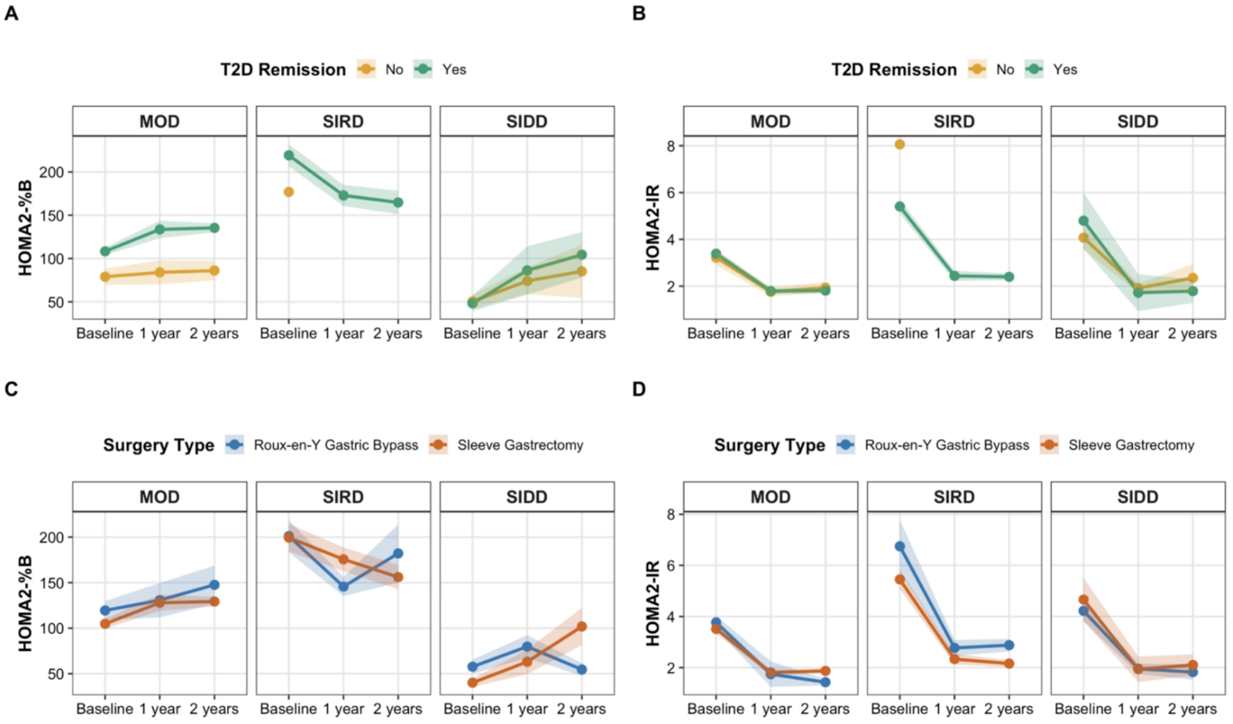

Supplementary Figure S2. HOMA2- indices over time by Subphenotype and T2D remission status (A, B) and type of surgery (C, D)

Abbreviations: HOMA2-%B, homeostatic model assessment 2 of beta-cell function; HOMA2-IR, homeostatic model assessment 2 of insulin resistance; MOD, mild obesity-related diabetes; SIDD, severe insulin-deficient diabetes; SIRD, severe insulin-resistant diabetes; T2D, type 2 diabetes

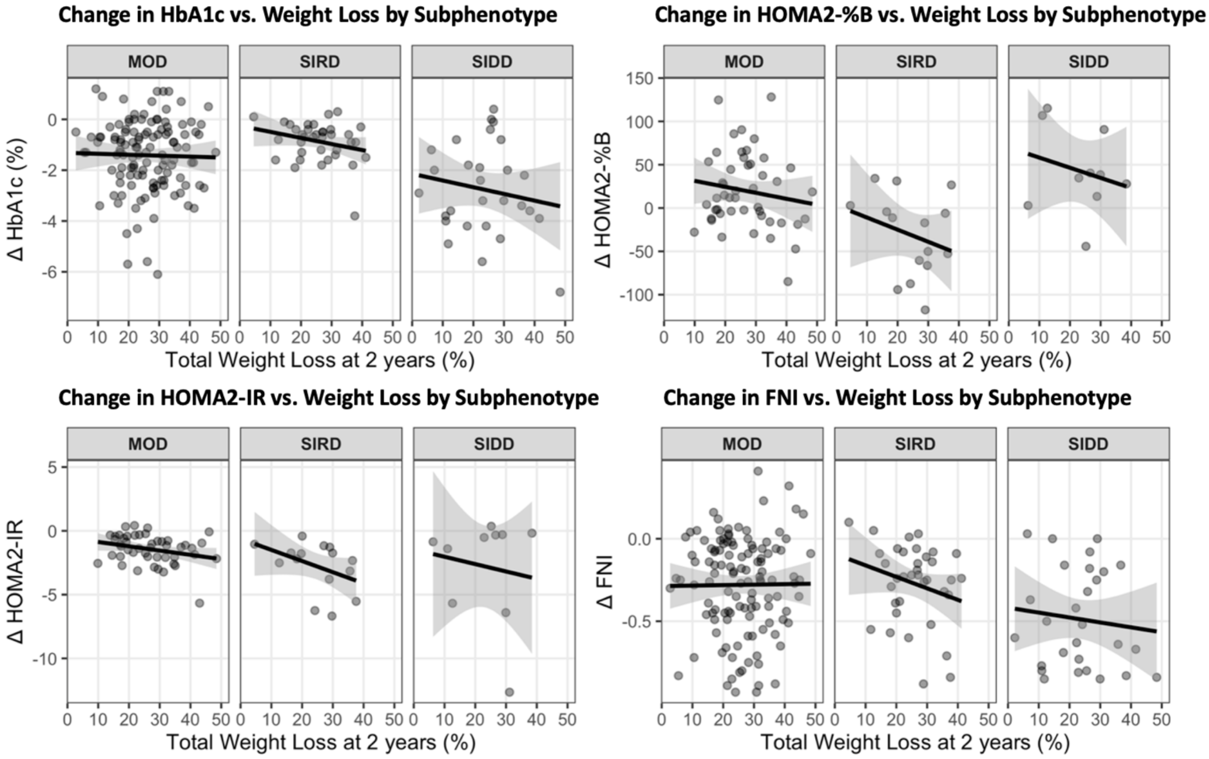

Supplementary Figure S3. Correlation between percent total weight loss at 2 years and changes in glycaemic and metabolic variables stratified by diabetes subphenotypes.

Lines represent linear regression fits; correlation coefficients were calculated using Spearman's rank correlation.

Abbreviations: FNI, Fibrotic NASH Index; HOMA2-%B, homeostatic model assessment 2 of beta-cell function; HOMA2-IR, homeostatic model assessment 2 of insulin resistance; MOD, mild obesity-related diabetes; SIDD, severe insulin-deficient diabetes; SIRD, severe insulin-resistant diabetes

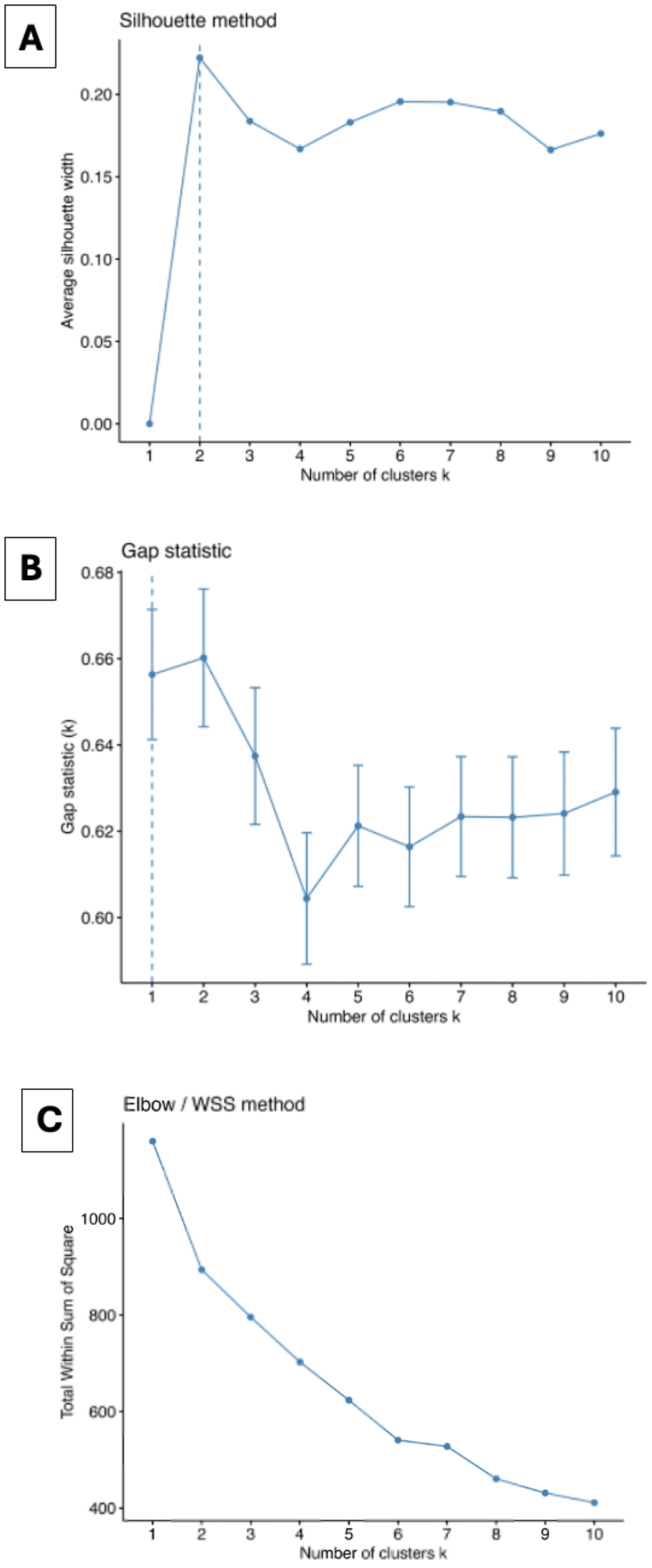

Supplementary Figure S4: Determination of the optimal number of clusters using the Silhouette width (A), gap statistic (B), and within-cluster sum of squares (elbow method)(C).

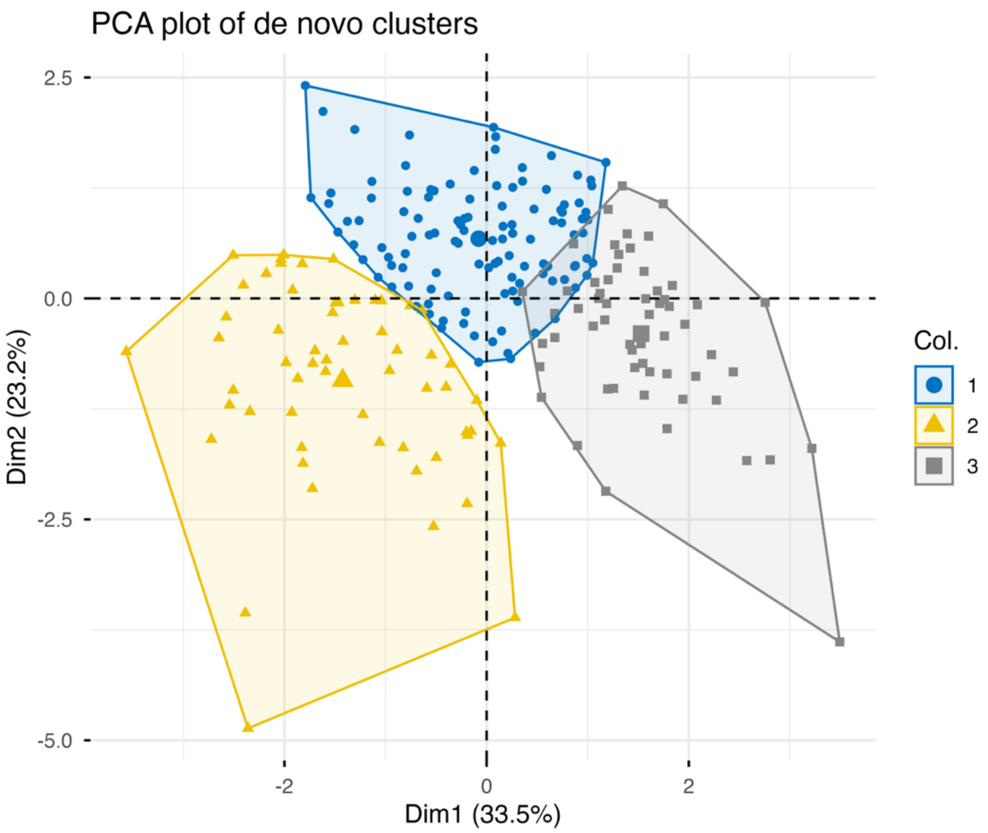

Supplementary Figure S5: Principal component analysis (PCA) of de novo clusters. PCA visualization demonstrates separation of the three de novo clusters based on scaled metabolic variables, with ellipses indicating cluster dispersion.

SUPPLEMENTARY MATERIAL

Supplementary Table S1: One-year outcomes based on T2D-subphenotypes

Variable	Overall N = 233[1]	MOD N = 145[1]	SIRD N = 46[1]	SIDD N = 42[1]	p-value[2]
**BMI (kg/m2)**					
1 year	31.7 ± 5.5	32.7 ± 5.3	31.6 ± 6.0	28.7 ± 4.6	**<0.001**
**HbA1c (%)**					
1 year	5.9 ± 1.0	5.7 ± 0.9	5.6 ± 0.6	7.0 ± 1.2	**<0.001**
**HOMA2-%B**					
1 year	120.9 ± 61.8	128.1 ± 61.1	167.1 ± 38.5	73.7 ± 43.8	**<0.001**
**HOMA2-IR**					
1 year	1.9 ± 0.8	1.8 ± 0.8	2.5 ± 0.7	2.0 ± 1.0	**0.01**
**T2D status at 1 year**					**<0.001**
Worsened	5 (2.2%)	2 (1.4%)	1 (2.3%)	2 (4.8%)	
Unchanged	5 (2.2%)	4 (2.9%)	1 (2.3%)	0 (0.0%)	
Improved	47 (21.1%)	21 (15.2%)	2 (4.6%)	24 (57.1%)	
Remission	166 (74.4%)	111 (80.4%)	39 (90.7%)	16 (38.1%)	
**%TWL**					
1 year	26.6 ± 8.1	27.0 ± 8.3	27.3 ± 6.8	24.6 ± 8.6	0.30
**Blood Platelets (G/L)**					
1 year	236.7 ± 71.5	243.2 ± 66.3	237.2 ± 65.1	214.8 ± 90.2	0.05
**AST (U/L)**					
1 year	23.0 ± 10.0	23.8 ± 11.3	22.9 ± 7.3	20.7 ± 7.4	0.14
**ALT (U/L)**					
1 year	25.7 ± 14.9	26.7 ± 16.8	23.1 ± 9.4	25.0 ± 12.4	0.80
**APRI-Score**					
1 year	0.3 ± 0.2	0.3 ± 0.2	0.3 ± 0.1	0.3 ± 0.1	0.70
**HDL (mg/dl)**					
1 year	54.2 ± 13.6	55.2 ± 14.6	54.1 ± 13.1	51.0 ± 10.1	0.30
**GFR (ml/min/1.73)**					
1 year	94.9 ± 18.4	94.0 ± 19.3	91.0 ± 15.6	102.2 ± 16.4	**0.02**
**Creatinine (mg/dl)**					
1 year	0.8 ± 0.2	0.8 ± 0.2	0.8 ± 0.2	0.7 ± 0.2	0.07

SUPPLEMENTARY MATERIAL

Variable	Overall N = 233[1]	MOD N = 145[1]	SIRD N = 46[1]	SIDD N = 42[1]	p-value[2]
**FNI**					
1 year	0.1 ± 0.1	0.1 ± 0.1	0.1 ± 0.1	0.2 ± 0.2	**0.03**

[1]Mean ± SD; n (%)

[2]Kruskal-Wallis rank sum test; Pearson's Chi-squared test

Abbreviations: ALT, alanine aminotransferase; APRI, aspartate aminotransferase to platelet ratio index; AST, aspartate aminotransferase; BMI, body mass index; FNI, Fibrotic NASH index; GFR, glomerular filtration rate; HOMA2-%B, homeostatic model assessment 2 of beta-cell function; HOMA2-IR, homeostatic model assessment 2 of insulin resistance; MASLD, metabolic dysfunction–associated steatotoc liver disease; MASH, metabolic dysfunction–associated steatohepatitis; MOD, mild obesity-related diabetes; NAS, NAFLD activity score; RYGB, Roux-en-Y gastric bypass; SG, sleeve gastrectomy; SIDD, severe insulin-deficient diabetes; SIRD, severe insulin-resistant diabetes; %TWL, percentage of total weight loss; T2D, type 2 diabetes;

Supplementary Table S2: Baseline diabetes medication by subphenotype

Drug_class	MOD (%)	SIRD (%)	SIDD (%)
DPP-4 inhibitor	0.5	1.7	3.7
GLP-1 RA	4.2	5.2	2.5
Insulin (any)	13.5	10.3	24.7
Metformin	53.5	58.6	37.0
Other	14.4	15.5	25.9
SGLT2 inhibitor	5.6	3.4	2.5
Sulfonylurea	8.4	5.2	3.7

Abbreviations: DPP-4. Dipeptidyl peptidase 4; GLP-1 RA, Glucagon-Like Peptide-1 receptor agonists, MOD, mild obesity-related diabetes; SGLT2, Sodium-glucose cotransporter 2; SIDD, severe insulin-deficient diabetes; SIRD, severe insulin-resistant diabetes;

Percentages represent the proportion of patients receiving each medication class; patients may be included in multiple categories due to combination therapy.

Supplementary Table S3: Number of diabetes medications and insulin use at baseline

Subphenotype	Number of drug classes	Insulin use (%)
MOD	1.5 (0.7)	20.0
SIRD	1.3 (0.6)	13.6
SIDD	2.0 (0.8)	48.8

Abbreviations: MOD, mild obesity-related diabetes; SIDD, severe insulin-deficient diabetes; SIRD, severe insulin-resistant diabetes;

Insulin use is reported as a patient-level variable, irrespective of concomitant glucose-lowering therapies.

Supplementary Table S4: Diabetes medications and insulin use at 2-year follow-up

Drug class	MOD (%)	SIRD (%)	SIDD (%)
No medication	119 (83.2%)	44 (95.7%)	21 (48.8%)
Oral antidiabetic drug	18 (12.6%)	2 (4.3%)	19 (44.2%)
Insulin	6 (4.2%)	0 (0.0%)	3 (7%)

Abbreviations: MOD, mild obesity-related diabetes; SIDD, severe insulin-deficient diabetes; SIRD, severe insulin-resistant diabetes;

Supplementary Table S5: HOMA2-indices by subphenotype and T2D remission status

Subphenotype	Variable	Timepoint	No T2D remission[1]	T2D remission[1]	p-value
**MOD (n=145)**	HOMA2-%B	Baseline	79.0 ± 46.8	108.3 ± 43.5	**0.002**
		1 year	84.0 ± 36.7	133.6 ± 61.3	**0.02**
		2 years	86.1 ± 24.9	135.4 ± 34.0	**0.001**
	HOMA2-IR	Baseline	3.2 ± 1.6	3.4 ± 1.5	0.50
		1 year	1.8 ± 0.5	1.8 ± 0.8	0.85
		2 years	1.9 ± 0.5	1.8 ± 0.6	0.47
**SIRD (n=46)**	HOMA2-%B	Baseline	176.9 ± NA	219.2 ± 74.8	0.56
		1 year	NaN ± NA	172.8 ± 40.4	-
		2 years	NaN ± NA	164.7 ± 52.6	-
	HOMA2-IR	Baseline	8.1 ± NA	5.4 ± 1.8	0.25
		1 year	NaN ± NA	2.4 ± 0.7	-
		2 years	NaN ± NA	2.4 ± 0.6	-
**SIDD (n=42)**	HOMA2-%B	Baseline	50.4 ± 35.2	48.2 ± 29.7	0.85
		1 year	74.1 ± 52.2	86.3 ± 48.1	0.45
		2 years	85.2 ± 61.3	104.4 ± 58.8	0.73
	HOMA2-IR	Baseline	4.1 ± 2.2	4.8 ± 4.0	0.90
		1 year	1.9 ± 0.7	1.7 ± 1.4	0.63
		2 years	2.4 ± 1.2	1.8 ± 1.1	0.54

[1]Mean ± SD; n (%)

Abbreviations: HOMA2-%B, homeostatic model assessment 2 of beta-cell function; HOMA2-IR, homeostatic model assessment 2 of insulin resistance; MOD, mild obesity-related diabetes; SIDD, severe insulin-deficient diabetes; SIRD, severe insulin-resistant diabetes; T2D, type 2 diabetes

Supplementary Table S6: HOMA2-indices by subphenotype and surgery type

Subphenotype	Variable	Timepoint	RYGB[1]	SG[1]	p-value
**MOD (n=145)**	HOMA2-%B	Baseline	119.5 ± 66.3	104.8 ± 54.3	0.26
		1 year	130.8 ± 37.8	127.9 ± 62.9	0.52
		2 years	147.6 ± 51.6	129.3 ± 33.7	0.27
	HOMA2-IR	Baseline	3.8 ± 1.7	3.5 ± 2.0	0.23
		1 year	1.7 ± 1.0	1.8 ± 0.8	0.50
		2 years	1.4 ± 0.3	1.9 ± 0.6	0.10
**SIRD (n=42)**	HOMA2-%B	Baseline	201.1 ± 79.0	199.5 ± 76.3	0.84
		1 year	145.7 ± 20.8	175.6 ± 41.4	0.14
		2 years	182.1 ± 71.4	156.1 ± 42.3	0.44
	HOMA2-IR	Baseline	6.7 ± 4.6	5.5 ± 1.9	0.56
		1 year	2.8 ± 0.6	2.3 ± 0.7	0.24
		2 years	2.9 ± 0.6	2.2 ± 0.4	**0.04**
**SIDD (n=42)**	HOMA2-%B	Baseline	57.7 ± 37.1	40.1 ± 19.8	0.12
		1 year	79.8 ± 46.8	63.1 ± 38.4	0.57
		2 years	54.6 ± 9.8	101.9 ± 57.7	0.40
	HOMA2-IR	Baseline	4.2 ± 1.9	4.7 ± 3.7	0.90
		1 year	2.0 ± 0.8	1.9 ± 1.4	0.54
		2 years	1.8 ± 0.4	2.1 ± 1.2	1.00

[1]Mean ± SD; n (%)

Abbreviations: HOMA2-%B, homeostatic model assessment 2 of beta-cell function; HOMA2-IR, homeostatic model assessment 2 of insulin resistance; MOD, mild obesity-related diabetes; RYGB, Roux-en-Y gastric bypass, SIDD, severe insulin-deficient diabetes; SIRD, severe insulin-resistant diabetes; SG, Sleeve gastrectomy, T2D, type 2 diabetes

Supplementary Table S7. Baseline Characteristics and Two-Year Outcomes Stratified by DiaSurg1 Status Within the SIDD Cluster

Characteristic	Overall N = 42[1]	Non-DiaSurg 1 N = 28[1]	DiaSurg 1 N = 14[1]	p-value[2]
**Sex**				>0.9
0	21 (50%)	14 (50%)	7 (50%)	
1	21 (50%)	14 (50%)	7 (50%)	
**Surgery**				<0.001
SG	19 (45%)	19 (68%)	0 (0%)	
RYGB	23 (55%)	9 (32%)	14 (100%)	
**Age_diagnosis**	43 ± 10	41 ± 11	46 ± 9	0.2
**Age_surgery**	54 ± 9	51 ± 9	58 ± 6	0.021
**Remission_2y**				0.10
No	19 (63%)	11 (52%)	8 (89%)	
Yes	11 (37%)	10 (48%)	1 (11%)	
**BMI_baseline**	38.3 ± 5.0	41.0 ± 3.6	32.8 ± 2.1	<0.001
**%TWL_2y**	24 ± 11	24 ± 13	26 ± 7	0.7
**HbA1c_baseline**	9.57 ± 1.66	10.16 ± 1.55	8.37 ± 1.17	0.001
**HbA1c_2y**	6.92 ± 1.12	6.68 ± 1.23	7.49 ± 0.56	0.044
**HOMA2-%B_baseline**	50 ± 31	39 ± 19	71 ± 40	0.009
**HOMA2-%B_2y**	92 ± 55	96 ± 57	62 ± NA	0.8
**HOMA2-IR_baseline**	4.42 ± 2.81	4.74 ± 3.28	3.78 ± 1.41	0.6
**HOMA2-IR_2y**	2.05 ± 1.05	2.04 ± 1.12	2.12 ± 1.2	0.8

[1]n (%); Mean (SD)

[2]Pearson's Chi-squared test; Wilcoxon rank sum test; Fisher's exact test; Wilcoxon rank sum exact test

Abbreviations: BMI, body mass index; HOMA2-%B, homeostatic model assessment 2 of beta-cell function; HOMA2-IR, homeostatic model assessment 2 of insulin resistance; RYGB, Roux-en-Y gastric bypass; SIDD, severe insulin-deficient diabetes; SG, sleeve gastrectomy; T2D, type 2 diabetes; %TWL, total weight loss

Supplementary Table S8: %TWL by subphenotype and surgery type

Subphenotype	%TWL	SG[1]	RYGB[1]	p-value
**MOD (n=145)**	1 year	26.9 (n=103)	27.5 (n=42)	0.72
	2 years	26.3	26.7	0.84
**SIDD (n=42)**	1 year	25.1 (n=19)	24.1 (n=23)	0.77
	2 years	24.2	24.7	0.91
**SIRD (n=46)**	1 year	26.8 (n=26)	27.9 (n=20)	0.59
	2 years	24.9	27.4	0.40

[1]Mean ± SD; n (%)

Abbreviations: MOD, mild obesity-related diabetes; RYGB, Roux-en-Y gastric bypass, SIDD, severe insulin-deficient diabetes; SIRD, severe insulin-resistant diabetes; SG, Sleeve gastrectomy, %TWL, percentage of total weight loss

Supplementary Table S9: Crosstable demonstrating concordance between original subphenotypes and de-novo subphenotype

De-novo subphenotype	Closest existing subphenotype	MOD	SIRD	SIDD	Composition (%)
**1**	MOD	66	2	5	90.4% MOD, 2.7% SIRD, 6.8% SIDD
**2**	SIDD	18	0	11	62.1% MOD, 0% SIRD, 37.9% SIDD
**3**	SIRD	12	16	0	42.9% MOD, 57.1% SIRD, 0% SIDD

Abbreviations: MOD, mild obesity-related diabetes; SIDD, severe insulin-deficient diabetes; SIRD, severe insulin-resistant diabetes

Supplementary Table S10: Metabolic characteristics of de-novo subphenotypes

Subphenotype_de_novo	n	Age_diagnosis	BMI_baseline	HbA1c_baseline	HOMA2_%B_baseline	HOMA2_IR_baseline
1	115	46.3	41.4	7.2	93.0	3.1
2	58	36.4	44.7	9.7	61.0	5.9
3	60	44.8	46.8	6.1	214.5	4.8

[1]Mean ± SD; n (%)

Abbreviations: BMI, body mass index; HOMA2-%B, homeostatic model assessment 2 of beta-cell function; HOMA2-IR, homeostatic model assessment 2 of insulin resistance;

Supplementary Table S11: Diabetes remission rates at two years between de-novo subphenotypes.

Suphenotype_de_novo	n	Remission at two years	p-Value (Chi2-test)
			0.001
1	70	59 (84.3%)	
2	26	14 (53.8%)	
3	24	22 (91.7%)	
